# Placenta-Related Parameters at Delivery in Relation to Folic Acid Supplementation in Different Pregnancies

**DOI:** 10.3390/nu16111729

**Published:** 2024-05-31

**Authors:** Yating Ren, Maoyuan Yang, Siyi Ren, Zhihao Ge, Yu Cao, Xinsheng Qin, Jie Sheng, Sufang Wang

**Affiliations:** 1 School of Public Health, Anhui Medical University, Hefei 230032, China; renyating97@163.com (Y.R.); yangmaoyuan@126.com (M.Y.); rensiyi2022@163.com (S.R.); 17754831523@163.com (Z.G.); 15374574557@163.com (Y.C.); qinxinsheng@ahmu.edu.cn (X.Q.); shengj@ahmu.edu.cn (J.S.); 2 Key Laboratory of Population Health Across Life Cycle, Ministry of Education of the People’s Republic of China, Hefei 230032, China; 3 NHC Key Laboratory of Study on Abnormal Gametes and Reproductive Tract, Hefei 230032, China

**Keywords:** folic acid, placenta, pregnancy, China

## Abstract

Folic acid plays an important role in the synthesis, repair, and methylation of deoxyribonucleic acid (DNA). Currently, most studies have focused on the effects of periconceptional folic acid (FA) supplementation on fetal development, and there is still a lack of population-based research exploring the association between FA use during pregnancy and placental development. This study aimed to investigate the impacts of FA supplementation in different pregnancies on placenta-related parameters at delivery. The study included 2708 pregnant women recruited from Ma’anshan City, Anhui Province, China, between May 2013 and September 2014. Information on FA use from one month before conception to delivery was collected. Placental length, width, and thickness were measured. Multivariable logistic regression analysis was used to assess the effects of FA supplementation in different pregnancies on placenta-related parameters. Based on multiple regression analysis, propensity score weighting was adopted to enhance comparability between different FA supplementation groups. Compared with FA non-users, FA supplementation before conception was associated with increased placental width (0.241 cm, 95%CI: 0.052–0.429, *p* = 0.013) and increased placental surface area (6.398 cm^2^, 95%CI: 1.407–11.389, *p* = 0.012), and FA use in early/middle pregnancy was, respectively, related with increased placental thickness (0.061 cm, 95%CI: 0.004–0.117, *p* = 0.036; 0.066 cm, 95%CI: 0.004–0.129, *p* = 0.038). FA use before conception could increase placental width and area, and FA use in early/middle pregnancy could increase placental thickness. To confirm the findings, further investigations are needed.

## 1. Introduction

Folic acid (FA), as a carrier of one-carbon units, plays a crucial role in one-carbon metabolism, especially in the conversion of homocysteine (Hcy) to methionine, promoting the synthesis of S-adenosylmethionine (SAM). SAM is the most important methyl donor in the human body; it participates in the methylation of deoxyribonucleic acid (DNA) and histones, and is closely related to the regulation of gene expression. In addition, FA is involved in de novo synthesis of DNA, converting deoxy uridine monophosphate (dUMP) to deoxythymidine monophosphate (dTMP) [1]. Insufficient FA in the body hinders the transfer of one-carbon units, affecting nucleic acid synthesis and amino acid metabolism, leading to impaired cell division and protein synthesis. This effect is particularly pronounced in rapidly dividing embryonic tissues [2]. Due to the rapid development of the fetus and placenta, as well as the enlargement of the uterus and increase in blood volume during pregnancy [3], FA requirement for pregnant women is five to ten times higher than for non-pregnant women, and reaches the maximal level in the last trimester of pregnancy, so the risk of FA deficiency for pregnant women is much higher than for women in general [4].

Conclusive evidence has indicated that periconceptional FA supplementation can greatly reduce the prevalence of neural tube defects (NTDs) [5]. Such evidence has led to a clear recommendation for women who are planning a pregnancy to take 400 µg FA/day from preconception until the end of the first trimester [6]. Based on the characteristics of fetal growth and development, maintaining a high folate level throughout the pregnancy is crucial. Continuous FA supplementation after the first trimester can increase serum folate levels in late pregnancy, reduce serum homocysteine levels, and improve umbilical cord blood folate levels [7]. The Society of Obstetricians and Gynaecologists of Canada (SOGC) guidelines recommend supplementing 0.4 mg FA per day in the entire pregnancy and suckling period [8].

The placenta is a temporary endocrine organ that facilitates gas, nutrient, and waste exchange between maternal and fetal compartments [9]. The trophoblast cells of the placenta are derived from the fetus, but they are in close contact with the decidua and blood vessels of the mother through the chorionic villi of the placenta [10]. Adaptations in placental nutrient transfer capacity to meet fetal growth demands depend on placental size and shape [11]. Human placental trophoblast culture has shown that FA is essential for several critical stages of placental development, including invasion of the trophoblast, formation of placental blood vessels, and secretion of matrix metalloproteinases [12]. FA deficiency leads to disruption of DNA methylation and normal epigenetic regulation, which can affect placental development and function, lead to changes in fetal programming, and increase the risk of some diseases in adulthood [13]. Several studies have found that FA supplementation is beneficial to reduce homocysteine levels, and homocysteine can induce placental endothelial cell damage, affect the growth and development of the placenta and fetus, and even induce preeclampsia [14,15]. Current studies on the effects of FA supplementation on placental development have been inconsistent. A survey found that pregnant women taking 5 mg of FA per day had an 11.9% average increase in placental weight compared to those not taking FA [16]. Some studies have shown that FA supplementation is not associated with placental weight [17,18]. It is well-known that placental weight is related to birth weight [19]. Therefore, when folic acid directly affects placental growth and development, it also indirectly impacts fetal growth. Placental weight is just one of the indicators of placental development and cannot fully explain the development of the placenta. This study attempts to comprehensively investigate the effects of FA supplementation in different pregnancies on placental development from multiple dimensions.

However, current research on the mechanisms behind birth defects caused by folate deficiency mainly focuses on fetal development itself. There is still a lack of population-based research exploring maternal–fetal interactions during pregnancy from this perspective. While FA supplementation is recommended throughout pregnancy in China, there are fewer pregnant women who use FA in middle and late pregnancy. Based on this situation, the innovation of this study is that we explored in depth the relationship between FA supplementation at different stages of pregnancy and placental parameters, including the length, width, area, and thickness of the placenta.

## 2. Materials and Methods

### 2.1. Study Population

**Figure 1:**
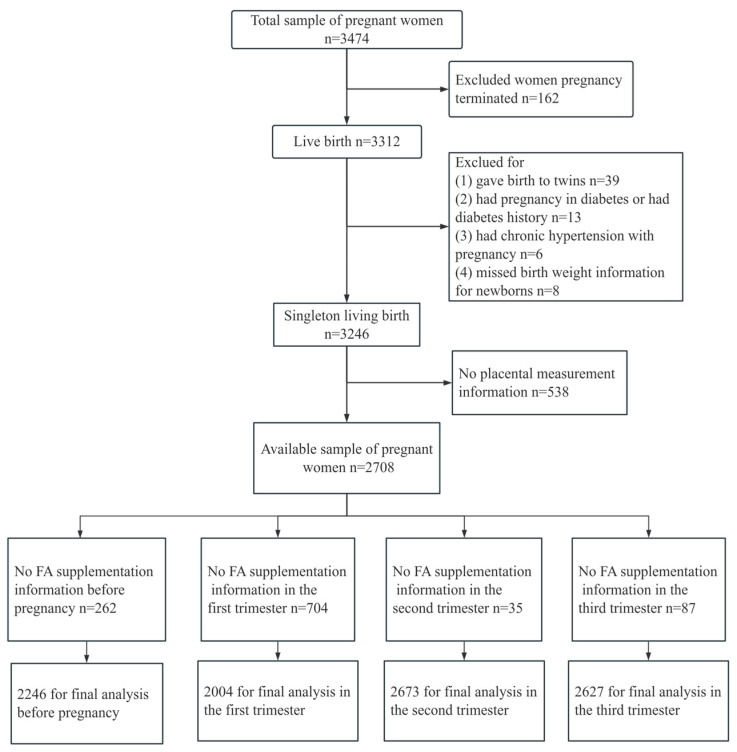
Flow diagram of recruitment and follow-up in this birth cohort study.

This study was based on the Ma’anshan Birth Cohort Study (MABC), which was a population-based longitudinal prospective study conducted in the Chinese city of Ma’anshan, in Anhui Province. Pregnant women were consecutively recruited at their first antenatal care visit in Ma’anshan Maternal and Child Health Care Center from May 2013 to September 2014 by trained investigators with the same inclusion criteria. For inclusion, participants had to fulfill the following criteria: age ≥ 18 years, gestational weeks < 14, with a singleton pregnancy. Eligible participants had no mental problems and sufficient understanding and expressive capacity, and lived in the Ma’anshan area for more than half a year with the intention to deliver at the reference hospital. A total of 3474 pregnant women were included in the cohort. Some participants were excluded because they had embryo damage (*n* = 162), a multiple pregnancy (*n* = 39), a diagnosis of pre-existing diabetes (*n* = 13), a hypertensive disorder complicating pregnancy (*n* = 6), missing birth weight (*n* = 8), resulting in a remaining total of 3246 single live birth mother–child pairs. In addition, in this study, 538 participants were excluded due to missing placenta measurements, and 2708 pregnant women had detailed placenta data at delivery. Among these participants, 262 pregnant women took complex FA or lacked FA supplementation data before pregnancy, 704 in the first trimester, 35 in the second trimester (*n* = 35), 81 in the third trimester, and were also excluded. Ultimately, 2446 pregnant women were included in the final analysis on the association between FA use before pregnancy and placenta-related parameters at delivery, and 2004 were included in the final analysis on the association between FA use in the first trimester and placenta-related parameters at delivery, with 2673 in the second trimester and 2627 in the third trimester (Figure 1).

### 2.2. Data Collection

Extensive data were collected using a structured self-report questionnaire that was administered by trained interviewers during each trimester. Basic data collected included age, race, education, social-economic status, place of residence, smoking, alcohol consumption, anthropometric measures, history of pregnancy and childbirth, history of diseases, etc.

### 2.3. Folic Acid Supplementation

To assess the information on the use of FA supplements in different periods (one month before pregnancy, first/second/third trimester of pregnancy), all participants were asked to complete a questionnaire independently at the time of their routine obstetric visit. The content of the questionnaire covered the following aspects: the date supplement use began, the frequency of use (times/week), and dose and brand names of supplements.

### 2.4. Measurements of Placental Size and Shape

Data on placental size and shape were measured after birth within 0.5 h by trained personnel. The placenta was expanded on a flat surface with the cotyledons facing upwards. The longest length of the placental surface was measured along with the maximal diameter using a transparent plastic ruler placed on it. The diameter perpendicular to the length was defined as the breadth. Placental thickness was measured at the thickest point. To calculate area, supposing the shape of the placenta is elliptical, the surface area of the placenta was defined as length × breadth × π/4.

### 2.5. Covariates

According to the standard of Working Group on Obesity in China, pre-pregnancy body mass index (BMI) was categorized into four groups: underweight (BMI < 18.5 kg/m^2^), normal weight (18.5 ≤ BMI < 24.0 kg/m^2^), overweight (24.0 ≤ BMI < 28.0 kg/m^2^), and obesity (BMI ≥ 28.0 kg/m^2^). Gestational Diabetes Mellitus (GDM) was diagnosed according to the American Diabetes Association (ADA) criteria [20]. Gestational hypertension was defined as having a systolic blood pressure ≥ 140 mmHg or a diastolic blood pressure ≥ 90 mmHg occurring after gestational week 20 [21].

### 2.6. Statistical Analysis

Analyses were performed using SPSS, version 16.0, and Stata, version 10.0 for Windows. Continuous variables were presented as means ± standard deviation and categorical variables were reported as frequencies (percent) [*n* (%)]. Categorical data (fetal gender, parity, maternal education, residence, income, occupation, race, delivery mode, cigarette smoking, and alcohol consumption) were analyzed using a chi-squared test, while continuous data (maternal age, pre-pregnant BMI, and gestational age) were analyzed by analysis of variance (ANOVA) or *t*-test. Multiple linear regression was used to analyze the effect of FA supplementation during pregnancy on placental parameters.

In addition, given the possible selective bias in receiving FA supplements, we performed a propensity score analysis to adjust for those factors related to selecting FA use in different pregnancies. Propensity scores were calculated using logistic regression with FA supplementation as the outcome variable and all available variables as predictors. Weighted logistic regression used the inverse of the propensity scores as weights to assess the effects of FA supplementation on placenta-related parameters. All statistical tests were performed at the two-sided 0.05 level of significance.

## 3. Results

### 3.1. Demographic Characteristics

The mean (SD) age of the 2708 pregnant women was 26.63 ± 3.8 years, with a pre-BMI of 20.8 ± 2.8 kg/m^2^, respectively. Most of the participants were of Han ethnicity (98.6%), had a higher educational level (80.5% above high school), lived in urban areas (77.8%), had a higher income (74.2% with monthly income above CNY 2500 per capita). Almost 90.0% of women reported that they were giving birth for the first time. Only 4.0% of the women smoked during pregnancy and 8.0% of the women drank during pregnancy (Table 1).

### 3.2. Folic Acid Supplementation in Different Pregnancies

A total of 2708 placentas were measured in this study. After excluding pregnant women supplemented with compound FA or lacking FA supplementation data, there were a total of 2446 pregnant women before pregnancy, 2004 pregnant women in early pregnancy, 2673 pregnant women in mid-pregnancy, and 2627 pregnant women in late pregnancy. FA use was highest in the first trimester (81.7%), followed by before conception (26.7), and fewer pregnant women took FA in the third trimester (14.2%) and the second trimester (8.9%) (Table 2).

### 3.3. Placenta-Related Parameters Compared According to FA Use in Different Pregnancies

We analyzed the effects of taking FA supplements in different periods of pregnancy on placenta-related parameters at delivery. Compared with FA non-users, FA users before conception had significantly greater width and area of the placenta (*p* < 0.01), and FA users in the second and third trimester had greater placental thickness (*p* < 0.05) (Table 3).

### 3.4. Association of FA Supplementation in Different Pregnancies with Placenta-Related Parameters

To elucidate the association between FA supplementation in different pregnancies with placenta-related parameters at delivery, we conducted multivariate linear regression analyses. After only adjusting for gestational weeks and fetal sex, compared with FA non-users, FA supplementation before conception was significantly associated with greater placental length, width, area, and thickness, and FA supplementation in the second and third trimesters was significantly associated with larger placental thickness. After further adjusting for age at conception, pre-pregnancy weight and height, education level, residence in the last six months, monthly income per capita, smoking and drinking status of pregnant women, GDM, and gestational hypertension, FA use before conception was significantly associated with greater placental length, width, and area, and FA use in the third trimester was significantly associated with larger placental thickness (Table 4).

After adjusting for propensity scores, FA supplement use before pregnancy was associated with increased placental width (0.241 cm, 95%CI: 0.052–0.429, *p* = 0.013) and increased placental surface area (6.398 cm^2^, 95%CI: 1.407–11.389, *p* = 0.012), and FA supplement use in early/middle pregnancy was, respectively, related with increased placental thickness (0.061 cm, 95%CI: 0.004–0.117, *p* = 0.036; 0.066 cm, 95%CI: 0.004–0.129, *p* = 0.038). In addition, there were two borderline statistical significances between FA use before conception and increased placental length (0.193 cm, 95%CI: −0.017, 0.404, *p* = 0.071), and FA supplementation in late pregnancy and increased placental thickness (0.052 cm, 95%CI: −0.004, 0.109, *p* = 0.067) (Table 5).

## 4. Discussion

As a key cofactor of one-carbon unit metabolism, folate is involved in DNA synthesis, repair, and methylation, especially for the rapid division and proliferation of embryonic cells during pregnancy [2]. Our findings showed that FA supplement use before pregnancy was significantly associated with increased placental width and increased placental surface areas compared with FA non-users, and this relation was strong or monotonic even after adjusting for propensity scores. FA supplement use in early/middle pregnancy was, respectively, associated with increased placental thickness. To our knowledge, this is the first study to examine the effects of FA supplementation in different pregnancies on the placenta-related parameters at delivery from the population-based perspective.

There are limited data about the association between FA supplementation and placental growth in different pregnancies. In an ex vivo study, placentas cultured under low-FA conditions were shown to increase apoptosis of human full-term placental primary trophoblast [22]. Animal studies have shown that FA supplementation before and during pregnancy increases placental and fetal weight, maximum placental diameter, junction, and labyrinth volume, and blood vessel density compared with dexamethasone supplementation alone [16]. This may be due to folic acid increasing the expression levels of vascular endothelial growth factor A (VEGF-A) and placental growth factor mRNA (PIGF mRNA), increasing blood vessel density, and thus improving fetal placental growth restriction. Another animal study found that excessive folic acid supplementation led to a significant increase in plasma homocysteine levels and a significant decrease in placental weight in female mice, suggesting that homocysteine is one of the factors affecting placental development [23]. A Danish survey found that pregnant women taking 5 mg of FA per day had an 11.9% average increase in placental weight compared to those not taking folate acid from the 23rd week [24]. A FA intervention study carried out in India found that compared with iron supplementation alone, iron and FA supplementation (500 μg/d) in the last 12~16 weeks of pregnancy could significantly increase placental weight, placental cell number, average total DNA content, and total protein content in each placenta [25]. The Generation R Study group found that low maternal FA concentration in the first trimester was associated with lower placental weight [26]. Meanwhile, two other studies reported that FA supplementation was not associated with placental weight. A systematic review and meta-analysis that analyzed data from four randomized controlled trials indicated no significant effect of total folate intake on placental weight [17]. The reasons for this inconsistency might be due to the limited sample sizes or the small number of the included studies. A mouse experiment showed that there was no significant difference in placental weight between the folate-deficient group (0 mg/kg) and the folate-supplemented group (2 mg/kg) [18]. The findings of randomized controlled trials have been less than conclusive, and the relative paucity of the collected reports suggested an urgent need to develop further high-quality studies. Placental weight is one of the measurement standards for evaluating placental growth and function, and it is a comprehensive summary of different aspects. In addition, morphological indicators of the placenta also include its length, width, thickness, surface area, etc., but detailed placental parameters are rarely reported in these studies. The above studies suggest that folic acid supplementation during pregnancy can affect placental development. Additionally, the impact on placental phenotype varies with the timing of maternal folic acid supplementation.

Our results are reliable from a biological perspective. The metabolisms of FA, methionine, and choline, which collectively form the one-carbon metabolism, have long been recognized for their association with fetal development. These metabolic byproducts serve as universal methyl donors required for DNA and histone methylation. DNA and histone methylation control chromatin structure, thereby regulating gene transcription [27]. Following zygote formation, epigenetic reprogramming occurs during early pregnancy. This process has the potential to impact various aspects of embryonic development and cellular differentiation [28]. Several studies have reported that insufficient intake of folate may lead to disruption of a carbon metabolic pathway and lead to abnormal levels of placental DNA methylation [29,30], which can affect fetal development [31]. Recent cohort studies have shown that prenatal vitamin use in the first month of pregnancy was associated with lower DNA methylation, particularly in the placenta [32]. Sarah et al. found that women who preconceptionally started taking a folic acid supplement not only had significantly larger newborns but larger placentas as well, compared to women who did not use folic acid [33]. Our findings showed that FA supplement use before pregnancy was significantly associated with increased placental width and increased placental surface area compared with FA non-users. This may be due to the fact that periconception folic acid supplementation may cause epigenetic modifications in the preimplantation embryo, which may result in increased placental and fetal growth patterns. However, at present, these speculations require further detailed study.

Maternal nutritional status can directly or indirectly affect embryonic development [34]. The length, width, thickness, and area of the placenta at delivery reflect the development of the placenta throughout pregnancy. The placenta develops completely in the early and middle stages of pregnancy, starting from the differentiation stage of the morula trophoblast. The development of the placenta involves complex structural and physiological recombination, including rapid proliferation, migration, invasion of mononucleated trophoblast cells into the maternal uterus, and spiral artery remodeling [35]. FA supplementation was found to increase the invasive potential of placental trophoblast by almost two-fold, which was associated with the decreased expression of tumor suppressor genes and tissue inhibitors of matrix metalloproteinases [36]. This also explains our research results, where pre-pregnancy supplementation of FA helps mothers achieve sufficient levels of folate, which is beneficial for overall placental development.

The development of placental blood vessels is an important factor in determining placental function [37]. Recently, it was reported that FA use during pregnancy results in placental metabolic and angiogenesis gene expression changes [38]. In vivo experiments suggested that maternal FA supplementation enhanced the expression of VEGF-A, significantly increased the vascular density of placental stroma, and improved the nutrient utilization of the fetus [39], and low folate concentrations were reported to increase placental vascular resistance [26]. The placenta is vulnerable to oxidative stress because of the high metabolic activity of placental cells. As a methyl donor in the homocysteine cycle, folate deficiency can lead to an increase in homocysteine levels in the blood [40]. Elevated levels of homocysteine contribute to the induction of oxidative stress by promoting the production of hydrogen peroxide and superoxide free radicals. These detrimental factors impair the functionality of placental vascular endothelium, hinder the process of villus angiogenesis [41,42,43], and the lower the denudation degree of the villi capillary bed, the thinner the placenta thickness. Our findings showed that FA supplement use in early/middle pregnancy was, respectively, associated with increased placental thickness compared with FA non-users. A randomized study showed that maintaining folic acid supplementation at a dose of 400 µg/day after the initial trimester of pregnancy led to a noteworthy rise in maternal red blood cell folate and cord blood folate levels, while also preventing the decrease in maternal serum folate concentrations [7]. This may be related to the lower homocysteine levels in the maternal serum during early to mid-pregnancy due to higher levels of folic acid, which is beneficial for the longitudinal growth of placental blood vessels, thereby increasing placental thickness. However, more research is needed to confirm this.

In this study, after adjusting for propensity scores, we did not observe any association between FA supplementation in the third trimester and placental parameters. This finding may be attributed to the establishment of placental surface morphology during the 8–12-week gestational period, as the placenta undergoes rapid growth in early pregnancy and reaches its full size during the second trimester [44]. Since blood vessels are well-developed in the third trimester, FA supplementation in the third trimester may have a greater impact on fetal development. Based on the MABC birth cohort, we previously reported that continued supplementation of 400 µg/day of FA during the second and third trimesters significantly promoted fetal development [45]. Given that fetal growth peaks in the latter half of pregnancy, increased folic acid supply during this phase may directly support enhanced cellular synthesis [46]. In this study, we further investigated the relationship between placental parameters and newborn birth indicators. The results showed that the development of the placenta significantly affects the growth and development of the fetus in utero. For every unit increase in the length, width, thickness, area, and amniotic fluid volume of the placenta, the birth weight, length, head circumference, and chest circumference of the newborn correspondingly increased by a certain value (results not shown). Similar to our findings, Freedman et al. reported that a reduction in surface area of 83 cm^2^ is associated with a 260.2 g reduction in birthweight (95%CI, 299.9 to 220.6), after adjustment for other features of placental morphology and covariates. Reduced placental thickness was also associated with lower birthweight [47]. Therefore, when folic acid directly affects placental growth and development, it also indirectly influences fetal growth. In the MABC birth cohort, only 26.7% of women started taking FA supplements before conception, with FA supplementation rate peaking at 81.7% in the first trimester. Our findings suggest that FA use before pregnancy is important for placental development to better prevent birth defects, promote fetal development, and maintain its subsequent health further. Therefore, ensuring that FA supplementation begins before pregnancy is a crucial measure.

Our study had several merits. First, this study is based on a large-sample cohort study and utilizes extensive and reliable data sources collected from face-to-face interviews and medical records. Second, we used propensity scores to adjust for confounding variables, greatly enhancing the reliability of our inferences. Finally, we comprehensively elucidated the impacts of FA supplementation during different stages of pregnancy on placenta-related parameters from a population perspective, with a high level of etiological evidence. Moreover, it is worth noting certain limitations of our study. First, the lack of dietary information collection renders us incapable of evaluating the effect of dietary folate intake. Second, the absence of data from similar studies in the literature pertaining to the same population type poses a constraint, calling for further validation of our conclusions. Lastly, potential interactions among multiple vitamins were not taken into account.

## 5. Conclusions

In conclusion, our study provided evidence that FA supplementation at different stages of pregnancy had a potential impact on placental development. FA supplementation before conception affected the width and area of the placenta, while FA supplementation in early to mid-pregnancy only affected the thickness of the placenta. Due to the limitations of our study, further research is needed to confirm our findings and elucidate the mechanisms underlying the observed associations.

## Figures and Tables

**Table 1:** Demographic characteristics of participants in the current birth cohort study.

Maternal Characteristics	Mean ± SD ^b^ or *n* (%)
Age (y)	26.3 ± 3.8
≤24	802 (29.6)
25~29	1436 (53.0)
≥30	470 (17.4)
Race	
Han ethnicity	2671 (98.6)
Others	37 (1.4)
Residence	
Urban	2107 (77.8)
Suburb	376 (13.9)
Rural	225 (8.3)
Education	
Junior high school and below	529 (19.5)
High school or college	1460 (54.0)
Bachelor’s degree or above	719 (26.5)
Monthly income	
<2500	698 (25.8)
2500–4000	1174 (43.3)
≥4000	836 (30.9)
BMI ^a^ (kg/m^2^)	20.8 ± 2.8
<18.5	526 (19.4)
18.5–23.9	1862 (68.8)
≥24	320 (11.8)
Parity	
Primipara	2438 (90.0)
Multipara	270 (10.0)
Smoking	
Yes	107 (4.0)
No	2601 (96.0)
Drinking	
Yes	216 (8.0)
No	2492 (92.0)

^a^ BMI before pregnancy; ^b^ SD: standard deviation.

**Table 2:** Status of FA supplements used in different periods of pregnancy.

FA Use	Pre-Pregnancy *n* = 2446	First Trimester *n* = 2004	Second Trimester *n* = 2673	Third Trimester *n* = 2627
Yes	653 (26.7)	1638 (81.7)	239 (8.9)	372 (14.2)
No	1793 (73.3)	366 (18.3)	2434 (91.1)	2255 (85.8)

**Table 3:** Placenta-related parameters compared according to FA use in different pregnancies.

Pregnancy	Length (cm)	Width (cm)	Area (cm^2^)	Thickness (cm)	Amniotic Fluid Volume (mL)
Before conception					
Nonusers	18.86 ± 2.11	16.50 ± 1.92	245.90 ± 48.70	2.33 ± 0.44	397.57 ± 157.20
FA users	19.03 ± 2.07	16.74 ± 1.88	252.10 ± 50.71	2.37 ± 0.46	410.67 ± 161.24
*t*	−1.835	−2.758	−2.753	−1.886	−1.867
*p*-value	0.067	0.006 **	0.006 **	0.060	0.062
First trimester					
Nonusers	18.90 ± 2.14	16.56 ± 1.92	247.37 ± 49.20	2.30 ± 0.45	396.05 ± 155.60
FA users	18.84 ± 2.04	16.59 ± 1.89	247.17 ± 49.33	2.34 ± 0.45	397.69 ± 155.65
*t*	0.558	−0.341	0.072	−1.451	−0.191
*p*-value	0.577	0.733	0.943	0.147	0.849
Second trimester					
Nonusers	18.89 ± 2.09	16.56 ± 1.88	247.33 ± 48.80	2.34 ± 0.45	403.73 ± 159.39
FA users	19.03 ± 2.01	16.67 ± 1.93	250.69 ± 48.20	2.40 ± 0.46	401.58 ± 161.58
*t*	−1.001	−0.827	−1.017	−2.007	0.205
*p*-value	0.317	0.408	0.309	0.045 **	0.838
Third trimester					
Nonusers	18.91 ± 2.10	16.55 ± 1.90	247.32 ± 49.01	2.339 ± 0.44	403.49 ± 159.31
FA users	19.00 ± 2.00	16.75 ± 1.90	251.44 ± 48.79	2.393 ± 0.48	403.21 ± 160.77
*t*	−0.731	−1.858	−1.503	−2.155	0.032
*p*-value	0.465	0.063	0.133	0.031 **	0.974

** *p* < 0.05.

**Table 4:** Multivariate linear regression analyses of placenta-related parameters and FA use in different pregnancies.

Pregnancy	Length (cm)	Width (cm)	Area (cm^2^)	Thickness (cm)	Amniotic Fluid Volume (mL)
model Ⅰ					
Before conception					
β	0.199	0.231	6.408	0.043	12.750
95%CI	(0.010, 0.389)	(0.059, 0.404)	(1.361, 9.181)	(0.003, 0.083)	(−1.089, 26.589)
*p*-value	0.039 **	0.009 **	0.005 **	0.035 **	0.071
First trimester					
β	−0.034	0.000	−0.382	0.041	2.290
95%CI	(−0.268, 0.200)	(−0.214, 0.215)	(−5.972, 5.207)	(−0.011, 0.092)	(−14.699, 19.280)
*p*-value	0.776	0.997	0.893	0.121	0.792
Second trimester					
β	0.172	0.113	3.897	0.063	−4.540
95%CI	(−0.106, 0.450)	(−0.138, 0.364)	(−2.584, 10.379)	(0.003, 0.123)	(−25.378, 16.299)
*p*-value	0.224	0.377	0.309	0.039 **	0.669
Third trimester					
β	0.066	0.185	3.607	0.052	−1.163
95%CI	(−0.165, 0.296)	(−0.025, 0.395)	(−1.786, 9.001)	(0.003, 0.102)	(−18.496, 16.170)
*p*-value	0.577	0.083	0.190	0.039 **	0.895
Model Ⅱ					
Before conception					
β	0.206	0.239	6.783	0.031	10.070
95%CI	(0.017, 0.395)	(0.067, 0.412)	(2.348, 11.217)	(−0.009, 0.071)	(−3.859, 23.998)
*p*-value	0.032 **	0.007 **	0.003 **	0.129	0.156
First trimester					
β	−0.033	0.011	−0.174	0.051	4.306
95%CI	(−0.267, 0.201)	(−0.205, 0.227)	(−5.785, 5.438)	(0.000, 0.103)	(−12.773, 21.385)
*p*-value	0.784	0.918	0.952	0.051	0.621
Second trimester					
β	0.149	0.066	2.555	0.050	−3.018
95%CI	(−0.129, 0.427)	(−0.189, 0.321)	(−3.993, 9.103)	(−0.011, 0.110)	(−25.248, 19.211)
*p*-value	0.293	0.610	0.444	0.108	0.790
Third trimester					
β	0.094	0.193	4.402	0.052	7.371
95%CI	(−0.135, 0.323)	(−0.017, 0.402)	(−0.970, 9.775)	(0.002, 0.101)	(−10.842, 25.584)
*p*-value	0.422	0.071	0.108	0.042 **	0.428

β, partial regression coefficient. With FA nonusers as control, FA use resulted in changes in placenta-related parameters. Model I adjusted the gestational age and fetal sex. Model II further adjusted the pregnant women’s age at pregnancy, pre-pregnancy weight and height, education level, residence in the past six months, monthly income per capita in the family, smoking and drinking status of pregnant women, GDM, and hypertension during pregnancy; ** *p* < 0.05.

**Table 5:** Multivariate linear regression analyses of placenta-related parameters and FA use in different pregnancies (adjusting for propensity scores).

Pregnancy	Length (cm)	Width (cm)	Area (cm^2^)	Thickness (cm)	Amniotic Fluid Volume (mL)
Before conception					
β	0.193	0.241	6.398	0.040	13.012
95%CI	(−0.017, 0.404)	(0.052, 0.429)	(1.407, 11.389)	(−0.011, 0.091)	(−4.092, 30.12)
*p*-value	0.071	0.013 **	0.012 **	0.128	0.136
First trimester					
β	0.021	−0.042	−0.541	0.061	17.687
95%CI	(−0.236, 0.278)	(−0.284, 0.201)	(−6.736, 5.654)	(0.004, 0.117)	(−0.550, 35.92)
*p*-value	0.873	0.737	0.864	0.036 **	0.057
Second trimester					
β	0.283	−0.006	3.507	0.066	−6.043
95%CI	(−0.027, 0.591)	(−0.343, 0.330)	(−4.563, 11.578)	(0.004, 0.129)	(−29.186, 17.10)
*p*-value	0.073	0.970	0.394	0.038 **	0.609
Third trimester					
β	0.120	0.172	4.096	0.052	6.115
95%CI	(−0.115, 0.355)	(−0.047, 0.392)	(−1.578, 9.770)	(−0.004, 0.109)	(−13.361, 25.59)
*p*-value	0.316	0.124	0.157	0.067	0.538

β, partial regression coefficient. With FA nonusers as control, FA use resulted in changes in placenta-related parameters. Adjusting for propensity score of fetal sex, gestational week, age before pregnancy, maternal height and weight before pregnancy, GDM, gestational hypertension, maternal smoking and alcohol consumption; ** *p* < 0.05.

## Data Availability

The data presented in this study are available upon request from the corresponding author. Due to their relevance to pregnant women, it is necessary to respect privacy and confidentiality.
